# The Effects of Recombinant pBD2 on the Growth Performance, Antioxidant Capacity, Immune Function, Intestinal Barrier, and Microbiota of Weaned Piglets

**DOI:** 10.3390/microorganisms13071443

**Published:** 2025-06-20

**Authors:** Zhanwei Teng, Qing Meng, Mengting Ren, Bingke Lv, Liping Yuan, Ningning Zhang, Qinghua Wang, Kun Zhang, Chunli Li

**Affiliations:** 1 College of Animal Science and Veterinary Medicine, Henan Institute of Science and Technology, Xinxiang 453003, China; 2 College of Animal Science and Technology, Henan Agricultural University, Zhengzhou 450046, China

**Keywords:** porcine β-defensin 2, antibiotic alternative, growth performance, intestinal barrier, intestinal microbiota

## Abstract

Defensins, one of the members of the antimicrobial peptide family, play a vital role in resisting microbial invasion and immune regulation. Porcine β-defensin 2 possesses excellent stability, making it an ideal antibiotic alternative for feed additives. In this study, a total of 15 piglets were used to investigate the effects of supplementing diets with 2.5 mg/kg (LP group) and 5 mg/kg (HP group) of pBD2 to weaned piglets. The results revealed that pBD2 significantly increased the total weight gain and average daily weight gain (*p* < 0.05), the contents of T-AOC, SOD, IgM, and IL-10 in serum (*p* < 0.05), the villus-to-crypt ratios, and the expression of tight-junction proteins ZO-1 and claudin-1 (*p* < 0.05) in the small intestine. Furthermore, pBD2 increased the abundance of beneficial bacteria related to nutrient and energy metabolism while decreasing the abundance of harmful bacteria associated with intestinal inflammation and diarrhea. Alterations in the gut microbiota were closely associated with the levels of T-AOC, SOD, IgM, and IL-10 in serum. pBD2 primarily enhanced the health of weaned piglets by influencing antioxidant capacity, intestinal barrier function, and the intestinal microbiota. Our research provides a novel perspective for addressing the issue of antibiotic residues in feed.

## 1. Introduction

Antibiotics are highly effective agents for disease resistance and growth promotion. However, the extensive and abusive use of antibiotics in animal husbandry has led to drug residues and the emergence of antibiotic-resistant bacteria that threaten human health and the environment. Thus, there is a consensus to reduce and ultimately ban the use of antibiotics in the livestock industry [1]. In 2020, China comprehensively banned the use of antibiotics as feed additives. In intensive culture, the growth rate slows down, and diseases become more difficult to prevent and control due to bacterial infection under antibiotic-free farming. More measures are being taken to offset the negative impact caused by the ban on antibiotic use; these measures mainly include improved management, the cultivation of new breeds, and nutritional intervention. Among these, nutritional intervention is the most direct and effective way to improve pigs’ disease resistance and productivity. Nutritional intervention involves adding nutrient substances, such as amino acids [2], as well as non-nutrient substances, such as probiotics [3], plant extracts [4], oligosaccharides [5], enzyme preparations, and antibacterial peptides [6], to the diet. These substances enhance animals’ disease resistance by essentially improving antioxidant capacity, immune performance, and gut microbiota balance.

Defensins, as important members of the antibacterial peptide family, possess both antibacterial and immunomodulatory effects [7,8], suggesting that defensins could also serve as feed additives to promote growth and disease resistance [9]. Currently, a total of 28 defensins have been discovered in pigs, all of which are β-defensins [10]. Among them, several defensins have shown antibacterial activity, immunity, antioxidation, autophagy, and apoptosis in vitro or in a mouse model [11,12,13,14]. However, few studies have focused on substituting antibiotics through the application of pBD2 as feed additives. Specifically, the mature pBD2 protein is a 37-amino-acid peptide with disulfide bonds and is distributed in the tongue, liver, kidneys, and intestines of pigs [15,16]. pBD2 possesses strong antibacterial activity, excellent water solubility, heat resistance, and low hemolytic activity [17]. Enterotoxigenic *Escherichia coli* infection induces the expression of pBD2 [18]. pBD2 attenuates inflammatory responses in IPEC-J2 cells against *Escherichia coli* by inhibiting the TLRs-NF-κB/MAPK signaling pathway [14,19]. Transgenic mice overexpressing pBD2 have exhibited enhanced resistance to salmonella [20], and transgenic pigs overexpressing pBD2 have exhibited enhanced resistance to *Glaesserella parasuis* and *Actinobacillus pleuropneumoniae* infection [21,22]. The use of pBD2, as a potentially ideal antibacterial growth-promoting feed additive for pigs in antibiotic-free farming, does not result in residuals or immunogenicity because it is a self-produced antibacterial peptide. Weaned piglets often experience deteriorated intestinal stability and low immunity due to changes in diet and the environment, making them susceptible to pathogenic bacterial infections, leading to diarrhea [23]. Synthesized pBD2 has been observed to increase feed intake and average daily weight gain (ADG) [24]. In previous studies, we expressed and obtained high-purity recombinant pBD2 protein in an inexpensive and efficient manner. Recombinant pBD2 can alleviate inflammatory responses and intestinal damage in IPEC-J2 cells infected with *Escherichia coli* [14,19]. However, the impact of recombinant pBD2 on weaned piglets remains unclear.

In this experiment, we hypothesized that dietary supplementation with pBD2 at 2.5 mg/kg and 5 mg/kg would improve growth, antioxidant status, immune responses, and intestinal barrier function by modulating the gut microbiota in weaned piglets. Our study assessed the application of pBD2 as a feed additive and provides a new strategy for healthy and sustainable development in livestock.

## 2. Materials and Methods

### 2.1. Experimental Design and Animal Feeding

This study was carried out on the basis of an agreement approved by the Animal Welfare and Ethics Committee of the Henan Institute of Science and Technology (approval no. LLSC2023030). The recombinant pBD2 used in this experiment was expressed and purified via affinity chromatography using BL21(DE3) pLysSpET30a-pBD2 strains constructed in our laboratory [17], with the same source as in our other previous experiments [14,19]. A total of 15 healthy weaned Large White piglets—all male and aged 28 days—were selected and randomly divided into three groups, with 5 piglets in each group. The control group was fed a regular diet without antibiotics. The low-dose pBD2 (LP) group received a basal diet supplemented with 2.5 mg/kg of pBD2, while the high-dose pBD2 (HP) group received a basal diet supplemented with 5 mg/kg of pBD2. The ingredients and nutrient level of the basal diets are shown in Table 1. During the feeding period, the pig pens (6 m^2^) were cleaned daily, disinfected regularly, and maintained with good ventilation at a temperature of 25–28 °C. The piglets had free access to food and water. The experiment lasted for 14 days.

### 2.2. Sample Collection

After the experiment, venous blood was collected from each piglet, centrifuged at 3000 rpm for 15 min to separate the serum, and stored at −80 °C for measuring immunoglobulins, cytokines, and antioxidant indicators. After the pigs were euthanized, their abdominal cavities were opened, and the duodenum, jejunum, and ileum were rapidly separated. A 2 cm segment from each region was fixed in 4% paraformaldehyde for tissue sectioning. Additionally, a 5 cm segment from the same region was placed in a cryotube and stored in liquid nitrogen for RNA extraction.

### 2.3. Growth Performance Measurement

The piglets were fasted and weighed at the beginning and end of the experiment to calculate the total weight gain and ADG. The average daily feed intake (ADFI) was calculated based on daily feed consumption records.

### 2.4. Antioxidant Indicator Measurement

Serum total antioxidant capacity (T-AOC), superoxide dismutase (SOD), and malondialdehyde (MDA) were measured using a kit purchased from Solarbio Technology Co., Ltd. (Beijing, China). The detection process followed the manufacturer’s instructions.

### 2.5. Enzyme-Linked Immunosorbent Assay (ELISA)

The concentrations of serum cytokines IL-2, IL-6, IL-10, and TNF-α and immunoglobulins IgA, IgM, and IgG were measured using an ELISA kit purchased from mmbio Co., Ltd. (Shanghai, China). The detection process followed the manufacturer’s instructions.

### 2.6. Morphology of Intestinal Tissues

Intestinal tissues were fixed with 4% paraformaldehyde and underwent repeated changes in the fixative every other day for a total of three days. The intestinal tissues were subjected to gradient dehydration using different concentrations of alcohol; then, the clearing agent xylene was used to replace the alcohol within the tissue blocks. The tissue blocks were infiltrated with melted paraffin. Once the paraffin had completely infiltrated the tissue blocks, they were embedded. The embedded wax blocks were fixed on a microtome and sliced into 4 μm sections. Xylene was used for dewaxing, followed by a series of alcohol solutions from high to low concentrations, ending with distilled water. The tissue sections were stained with hematoxylin and eosin and then sealed with resin. The tissue slices were observed and photographed using a microscope, and the villus-to-crypt ratio was measured.

### 2.7. RNA Extract

The TRIzon kit was used to extract RNA according to the manufacturer’s instructions (Solarbio, Beijing, China). Briefly, a few intestinal tissues were ground into a powder with liquid nitrogen. About 100 mg of the tissue powder was dissolved in 1 mL of TRIzon in a 1.5 mL tube. The tube was shaken up and down to fully lyse the sample and then left at room temperature for 5 min to completely separate the nucleic acid–protein complexes. A total of 0.2 mL of chloroform was added to denature the proteins, followed by centrifugation at 12,000 rpm at 4 °C for 15 min. The upper aqueous phase was taken and transferred to a new centrifuge tube, and then an equal volume of isopropanol was added to make the nucleic acids precipitate. The nucleic acids were washed with 75% ethanol and dissolved thoroughly in water.

### 2.8. qPCR

Primers were designed using the Primier Premier 5.0 software based on the mRNA sequences in the gene bank and synthesized by Sangon Biotech Co., Ltd. (Shanghai, China) (Table 2). The RNA was converted to complementary (c) DNA according to the instructions of the reverse transcription kit of Yi sheng Biotechnology Co., Ltd. (Shanghai, China) (11141ES60). qPCR assays were performed in a quantitative real-time PCR cycler (LightCycler 96, Roche, Basel, Switzerland) according to a SYBR Green Dye kit (Vazyme, Nanjing, China). The PCR program was set to 95 °C for 30 s, followed by 40 cycles at 95 °C for 15 s, 60 °C for 30 s, and 72 °C for 15 s. The results were analyzed using the 2^−ΔΔCt^ method [25].

### 2.9. Microbiome Diversity Analysis

The colon was also separated, and the intestinal contents were collected in 10 mL cryotubes. The total DNA of the intestinal bacteria was extracted using a bacterial DNA kit, and the concentration of the DNA was measured using a NanoDrop 1000 spectrophotometer (Thermo Fisher Scientific, Waltham, MA, USA). The primers were designed based on the conserved regions of bacteria, and sequencing adapters were added to the ends of the primers. Subsequently, PCR amplification was performed targeting the V3 and V4 regions of the 16S rRNA gene, with primer sequences 338F (5′-ACTCCTACGGGAGGCAGCAG-3′) and 806R (5′-GGACTACHVGGGTWTCTAAT-3′) [26]. The PCR products were subjected to purification, quantification, and normalization processes to prepare a sequencing amplification library. The sequencing process was completed at Biomarker Technologies Co., Ltd. (Beijing, China), using the Illumina HiSeq 2500 platform (Illumina, San Diego, CA, USA).

The α-diversity indices of the gut microbiota in the piglets were analyzed using the Mothur software (version v.1.30). Chao1 and Ace were used to measure the species richness, that is, the number of species. The Shannon and Simpson indices were used to measure the species diversity. PCoA (principal coordinate analysis) was performed to analyze the β-diversity of the bacteria. The unweighted unifrac method was adopted to calculate the distances between samples, comparing the similarity of species diversity among different samples. LEfSe (linear discriminant analysis effect size) analysis was conducted to identify differentially abundant bacteria [27], and the minimal threshold of linear discriminant analysis (LDA) was set at 3.5. Pearson’s correlation coefficients were used to analyze the correlations of microbiota, immunoglobulins, cytokines, and the antioxidant index.

### 2.10. Statistical Analysis

The data were characterized as means and standard errors and assessed using one-way ANOVA followed by Duncan’s post hoc test in the SPSS 22 software (IBM, Armonk, NY, USA). *p* < 0.05 was considered statistically significant; *p* > 0.05 indicated non-significance.

## 3. Results

### 3.1. Effects of pBD2 on Growth Performance of Weaned Piglets

pBD2 significantly affected the total weight gain and ADG of weaned piglets (*p* < 0.05) and had no effect on the ADFI or FCR (feed conversion ratio) (*p* > 0.05). Compared with the control group, both the LP and HP groups significantly increased the total weight gain and ADG (*p* < 0.05), while there was no significant difference between the LP and HP groups (*p* > 0.05) (Table 3).

### 3.2. Effects of pBD2 on Antioxidant Capacity in Weaned Piglets

pBD2 significantly enhanced the antioxidant capacity of weaned piglets and had a significant effect on the serum T-AOC levels in weaned piglets (*p* < 0.05). There was no significant difference between the LP and HP groups. The SOD levels in the LP group were significantly higher than those in the control and HP groups (*p* < 0.05), while there was no significant difference between the HP and control groups (*p* > 0.05). pBD2 had no significant impact on MDA (*p* > 0.05) (Table 4).

### 3.3. Effects of pBD2 on Immune Performance in Weaned Piglets

To study the effect of pBD2 on the immune performance of weaned piglets, anti-inflammatory cytokines IL-10 and IL-2, pro-inflammatory cytokines TNF-α and IL-6, and immunoglobulins IgA, IgM, and IgG in serum were detected using the ELISA method. The HP group showed a significant increase in the content of the anti-inflammatory cytokine IL-10 (*p* < 0.05), while the LP group exhibited no significant difference compared with the control group (*p* > 0.05). There was no significant difference between the LP and HP groups (*p* > 0.05). pBD2 showed a trend of an increased IL-2 content compared with the control group (*p* = 0.066) but had no significant impact on the levels of pro-inflammatory cytokines TNF-α and IL-6 (*p* > 0.05). The IgM content in the HP and LP groups was significantly higher than that in the control group (*p* < 0.05), and there was no significant difference between the HP and LP groups (*p* > 0.05). pBD2 had no significant effect on the levels of immunoglobulins IgA and IgG (*p* > 0.05) (Table 5).

### 3.4. Effect of pBD2 on Intestinal Barrier Function in Weaned Piglets

**Figure 1:**
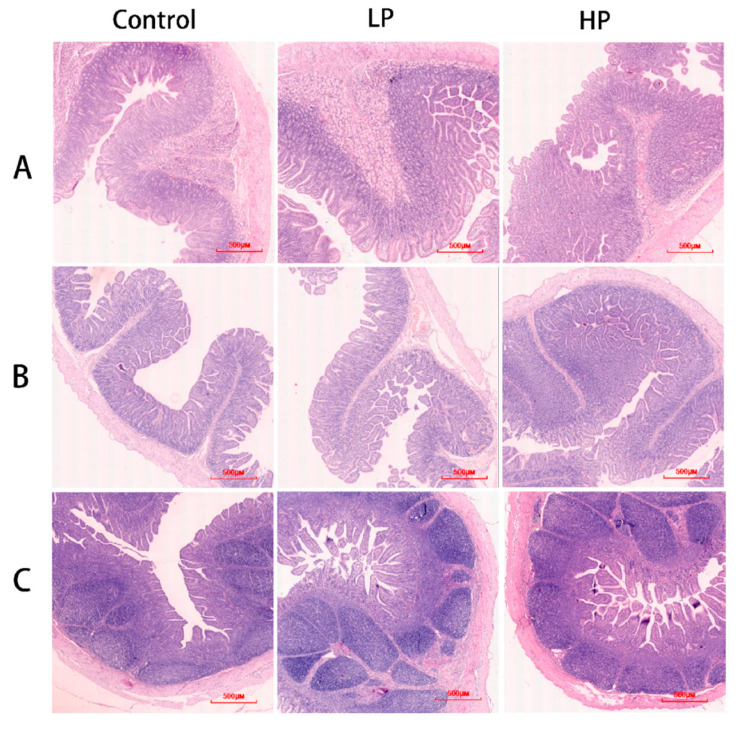
Morphology of intestine. Control, regular diet. LP, basal diet supplemented with 2.5 mg/kg of pBD2. HP, basal diet supplemented with 5 mg/kg of pBD2. (**A**–**C**) indicate duodenum, jejunum, and ileum, respectively.

**Figure 2:**
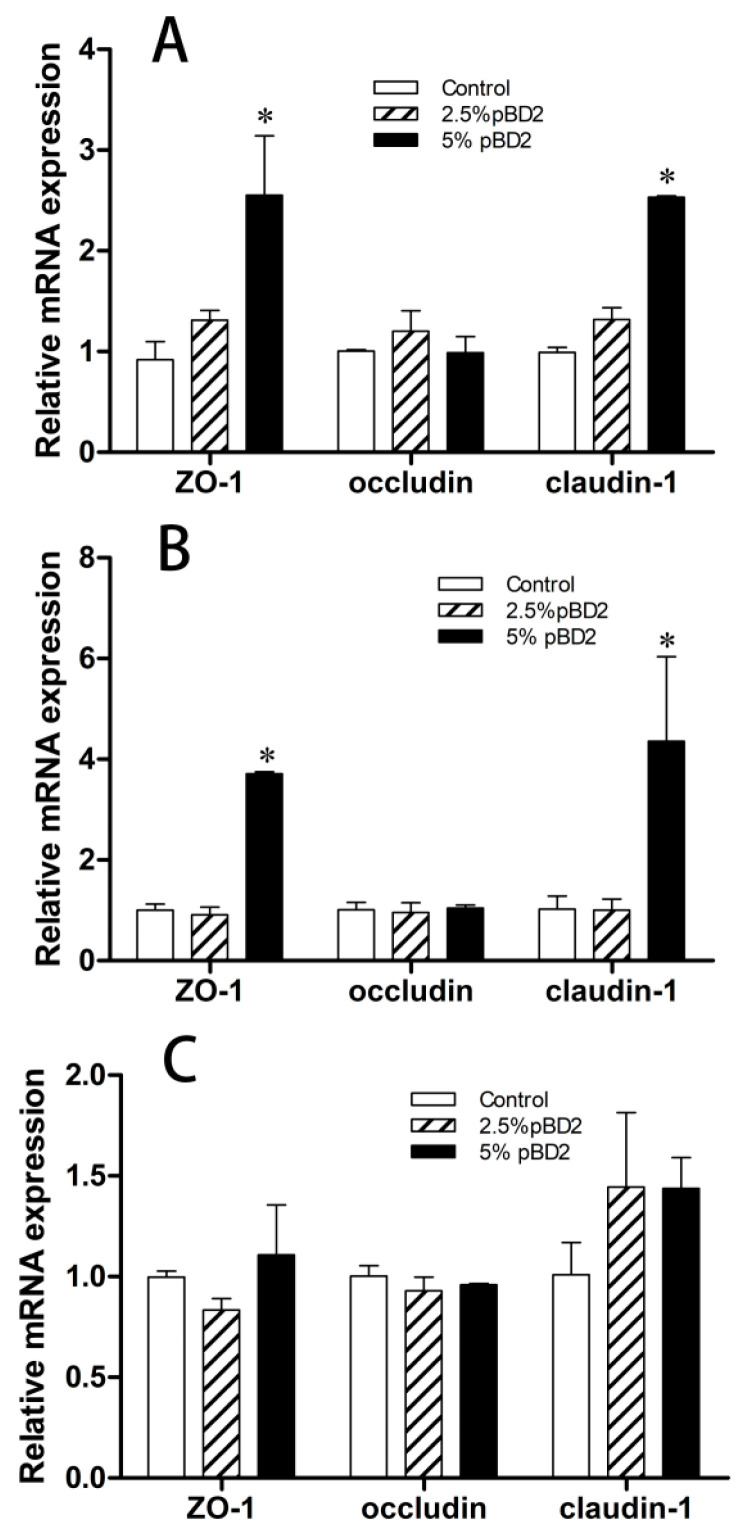
Effect of pBD2 on expression of tight-junction proteins in duodenum, jejunum, and ileum of weaned piglets. (**A**–**C**) indicate duodenum, jejunum, and ileum, respectively. Data are means ± SDs; n = 3. * indicates a significant difference compared with the control group at *p* < 0.05.

To investigate the effect of pBD2 on the intestinal barrier function of weaned piglets, the intestinal morphology, villus-to-crypt ratio, and expression of tight-junction proteins were analyzed. The results showed that pBD2 had no significant effect on the morphology of the duodenum, jejunum, and ileum and did not cause intestinal damage or inflammatory responses (Figure 1). In the duodenum, jejunum, and ileum, pBD2 had significantly higher villus-to-crypt ratios compared with the control group (*p* < 0.05). There was no significant difference between the LP and HP groups in the duodenum and jejunum (*p* > 0.05), but the HP group had a significantly higher villus-to-crypt ratio in the ileum compared with the LP group (*p* < 0.05) (Table 6). Furthermore, the HP group showed a significant increase in the expression of tight-junction proteins ZO-1 and claudin-1 in the duodenum and jejunum, with no significant change in the expression of occludin. LP had no significant effect on the expression of tight-junction proteins in the three intestinal segments (Figure 2).

### 3.5. Effects of pBD2 on Microbial Composition

**Figure 3:**
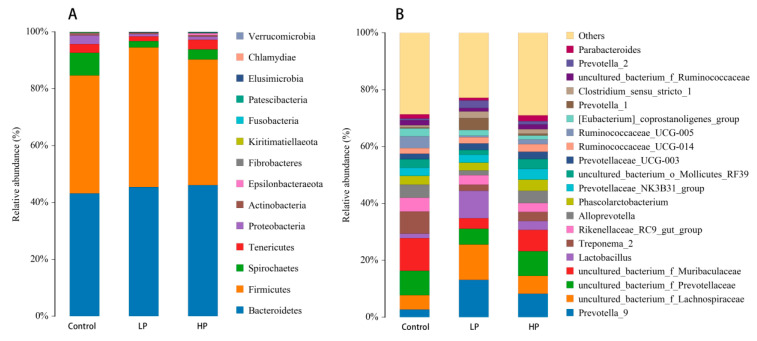
Microbial structure. (**A**,**B**) indicate the phylum and genus levels, respectively. Control, regular diet. LP, basal diet supplemented with 2.5 mg/kg of pBD2. HP, basal diet supplemented with 5 mg/kg of pBD2.

At the phylum level, a total of 14 phyla were identified in both the control and HP groups, while only 13 phyla were found in the LP group (without Chlamydiae). The relative abundance of major microbial taxa had changed. In the control group, the top four most abundant phyla were, in order, *Bacteroidetes*, *Firmicutes*, *Spirochaetes*, and *Proteobacteria*. In the LP group, the top four abundant phyla were *Firmicutes*, *Bacteroidetes*, *Spirochaetes*, and *Tenericutes*. In the HP group, the top four abundant phyla were *Bacteroidetes*, *Firmicutes*, *Spirochaetes*, and *Tenericutes* (Figure 3A).

At the genus level, the top four most abundant genera in the control group were, in order, uncultured_bacterium_f_*Muribaculaceae*, uncultured_bacterium_f_*Prevotellaceae*, T*reponema_2*, and uncultured_bacterium_f_*Lachnospiraceae*. In the LP group, the top four abundant genera were *Prevotella_9*, uncultured_bacterium_f_*Lachnospiraceae*, *Lactobacillus*, and uncultured_bacterium_f_*Prevotellaceae*. In the HP group, the top four abundant genera were uncultured_bacterium_f_*Prevotellaceae*, *Prevotella_*9, uncultured_bacterium_f_*Muribaculaceae*, and uncultured_bacterium_f_*Lachnospiraceae* (Figure 3B).

### 3.6. Effects of pBD2 on Alpha and Beta Diversity

**Figure 4:**
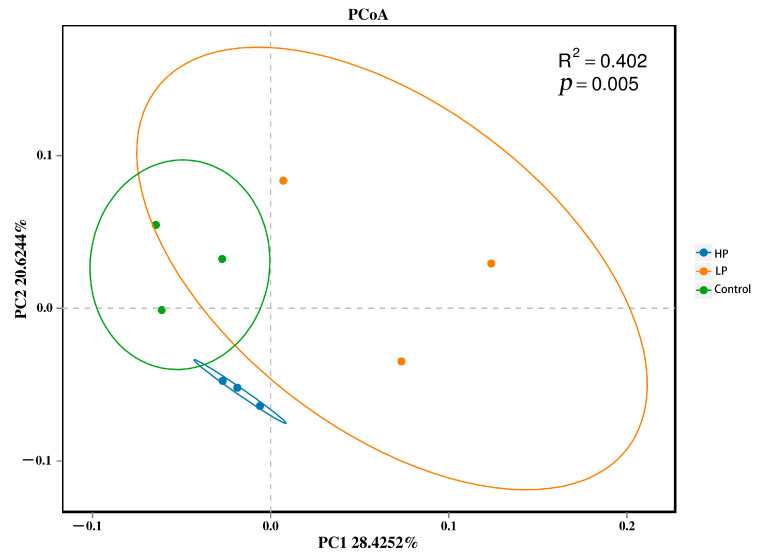
Analysis of beta diversity. Green, orange, and blue indicate the control, LP, and HP groups, respectively. Control, regular diet. LP, basal diet supplemented with 2.5 mg/kg of pBD2. HP, basal diet supplemented with 5 mg/kg of pBD2.

Alpha diversity analysis showed that the ACE index in the HP group was lower than that in the control group and not significantly different from that in the LP group. The ACE index did not significantly differ between the LP and control groups. There was no significant difference in the Chao1 index among the three groups. The Shannon and Simpson indices in the control and LP groups were significantly higher than those in the HP group, while there was no significant difference between the control and LP groups (Table 7). β-diversity analysis showed that the samples of different groups had significant differences (Figure 4). The above results indicate that pBD2 affected the diversity of the intestinal microflora.

### 3.7. Effect of pBD2 on Microbial Abundance

**Figure 5:**
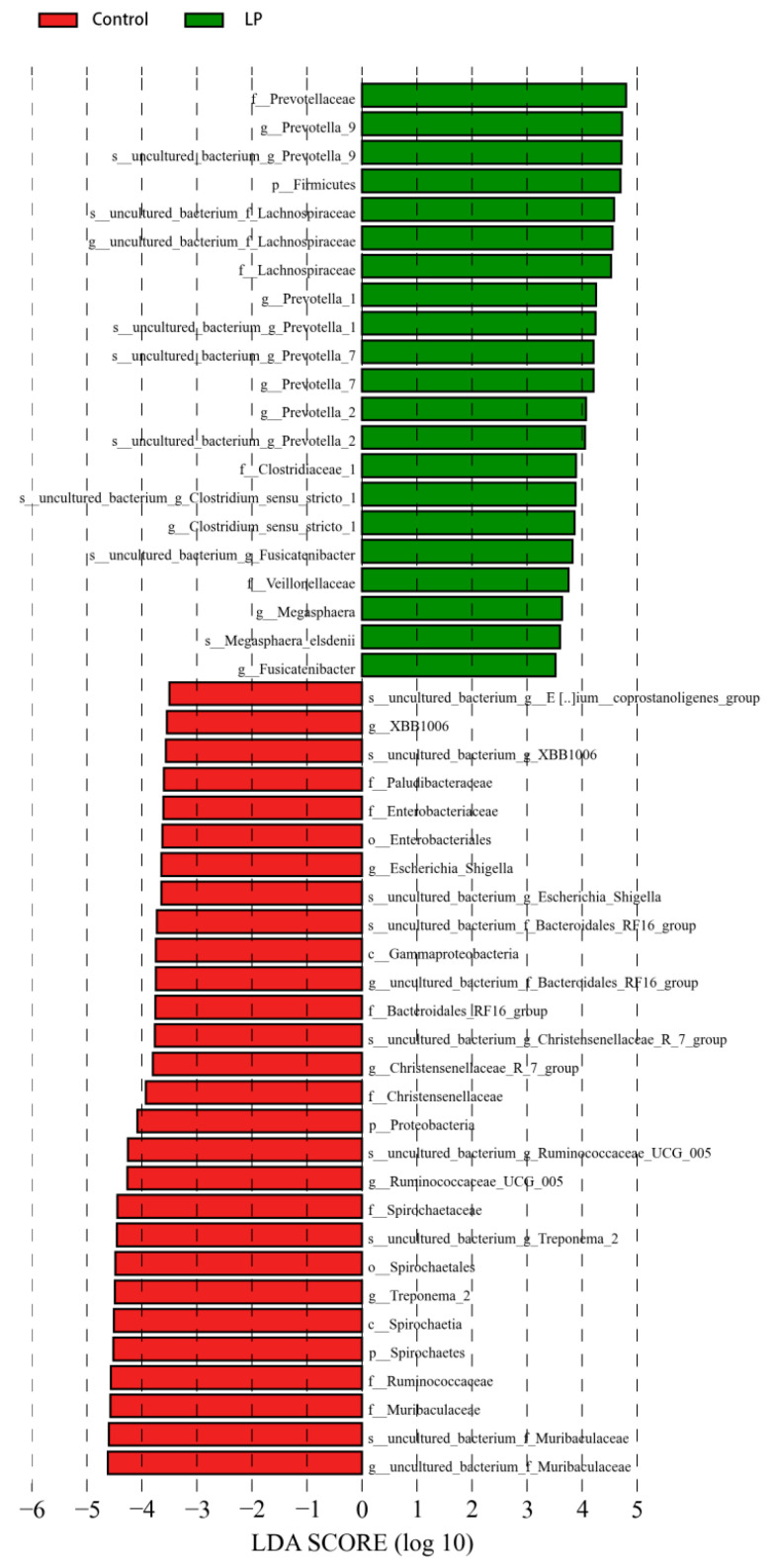
Histogram of LDA value distribution in LP and control groups. Red and green indicate control and LP groups, respectively.

**Figure 6:**
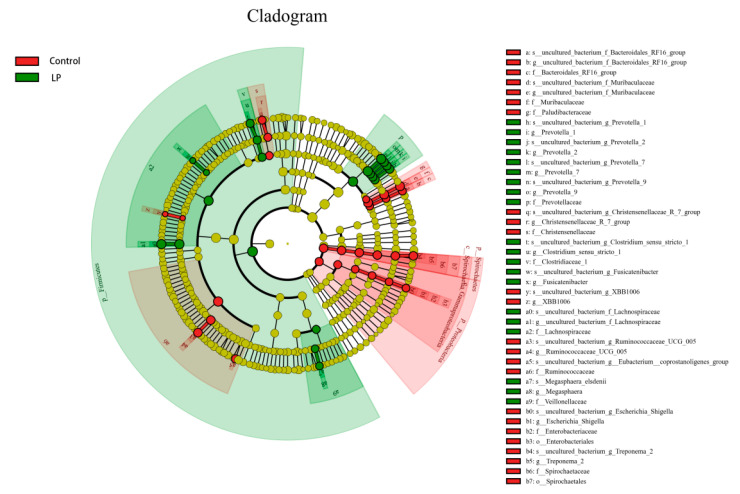
Evolutionary branching diagram in LP and control groups. Red and green indicate control and LP groups, respectively.

LEfSe was used to analyze bacterial abundance in the pBD2 treatment group and control group, with LDA > 3.5 as the screening threshold. In the LP group, the abundance of *Prevotella_*9, *Prevotella_*1, *Prevotella_*7, *Prevotella_*2, *Firmicutes, Lachnospiraceae, Clostridium_sensu_stricto_*1, *Fusicatenibacter*, *Veillonellaceae*, and *Megasphaera* was increased. The abundance of uncultured_bacterium_g_*Bacteroidales_RF*16*_group*, *Muribaculaceae*, *Paludibacteraceae*, *Christensenellaceae*, uncultured_bacterium_g_*XBB*1006, *Ruminococcaceae_UCG_*005, *Eubacterium-coprostanoligens_group*, *Escherichia_Shigella*, *Treponema* 2, and *Spirochaetaceae* was decreased (Figure 5 and Figure 6).

**Figure 7:**
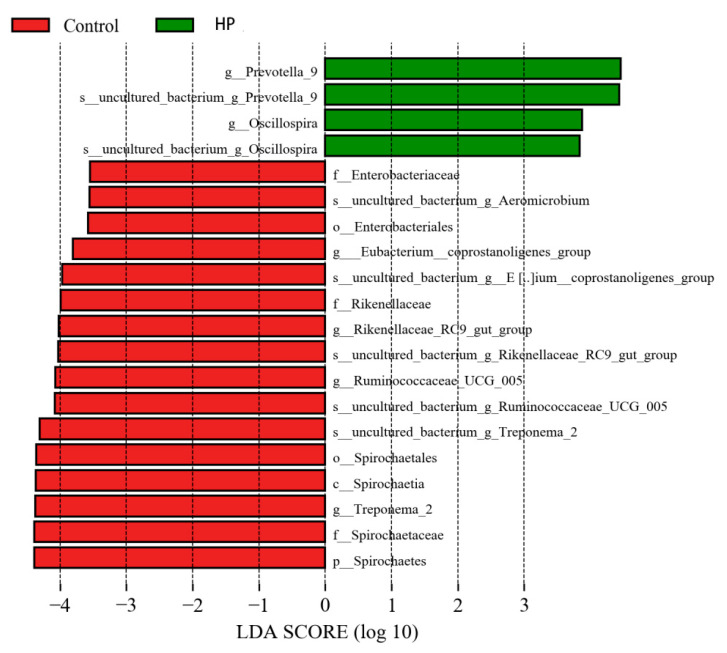
Histogram of LDA value distribution in HP and control groups. Red and green indicate control and HP groups, respectively.

**Figure 8:**
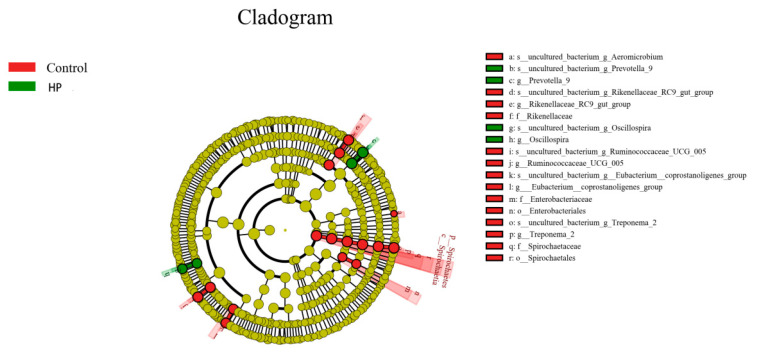
Evolutionary branching diagram in HP and control groups. Red and green indicate control and HP groups, respectively.

In the HP group, the abundance of Oscillospira and Prevotellaceae was increased, and that of Spirochaeta, Treponema 2, Ruminococcaceae_UCG_005, Rikenellaceae RC9_gut_group, Eubacterium-coprostanoligenes-group, and Mitsuokella was decreased (Figure 7 and Figure 8).

### 3.8. Correlation Analysis of Microorganisms with Immune Performance and Antioxidant Capacity

**Figure 9:**
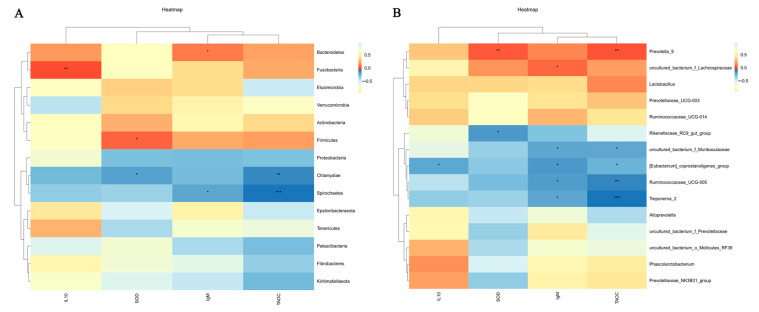
Correlation analysis of microorganisms and IgM, IL-10, SOD, and T-AOC. (**A**,**B**) indicate the top 15 phyla and genera in abundance for bacteria. Red indicates positive correlation; blue indicates negative correlation. * indicates *p* < 0.05; ** indicates *p* < 0.01; *** indicates *p* < 0.001.

Correlations were detected between significantly different cytokines and antioxidant indices (IgM, IL-10, SOD, and T-AOC) and the gut microbiota. At the phylum level, *Bacteroidetes* had a positive correlation with IgM; *Fusobacteria* had a positive correlation with IL-10; *Firmicutes* had a positive correlation with SOD; *Chlamydiae* had a negative correlation with SOD and T-AOC; and *Spirochaetes* had a negative correlation with T-AOC (Figure 9A). At the genus level, *Prevotella_*9 had a positive correlation with SOD and T-AOC; uncultured_bacterium_f_*Lachnospiraceae* had a positive correlation with IgM; *Rikenellaceae_RC*9*_gut_group* had a negative correlation with SOD; uncultured_bacterium_f_*Muribaculaceae*, *Ruminococcaceae_UCG-005*, and *Treponema_*2 had a negative correlation with IgM and T-AOC; and *[Eubacterium]_coprostanoligenes_group* had a negative correlation with IL-10, IgM, and T-AOC (Figure 9B).

## 4. Discussion

During weaning, piglets’ digestive systems are not fully developed, resulting in relatively weak digestive capabilities. The transition from breast milk to solid feed and the rearing environment may cause stress responses, intestinal injury, gut microbiota imbalance, and immune system weakening, which easily leads to bacterial infection and diarrhea [28]. Antibiotics are widely used to prevent bacterial infections, promote growth, and reduce diarrhea in weaned piglets. However, they have been banned as feed additives due to concerns about drug residue and antibiotic resistance.

Antibacterial peptides have unique bactericidal mechanisms that make it difficult for microorganisms to develop drug resistance [29,30]. Multiple studies have shown that AMPS also have excellent effects on disease resistance and growth promotion, making them ideal alternatives to antibiotics [6]. In this study, the purity of recombinant pBD2 was high, above 95%, meaning it is safe, efficient, and reliable. Dietary supplementation with pBD2 increased the total weight gain and average daily gain of weaned piglets but had no significant effect on food intake. It has been reported that dietary supplementation with 60 mg/kg AMP-P5 has the potential to improve the growth performance and apparent total tract digestibility of nutrients and reduce coliforms in weanling pigs [31]. Moreover, 0.5% crudely extracted pBD2 has been observed to significantly improve food intake and daily weight gain and reduce diarrhea rates [32], and 0.1 mg/mL synthesized pBD2 improves food intake and daily weight gain, with similar impacts observed with 0.6 mg/mL neomycin sulfate as well [24]. Our results are consistent with previous studies. Longer experimental phases and more concentration gradients may be required to further determine the effects of pBD2 as a feed additive.

Weaned piglets are prone to oxidative stress reactions during weaning. Oxidative stress can lead to intestinal inflammation and injury in weaned piglets. The level of MDA content reflects the degree of tissue peroxidation and cell damage [33]. SOD can specifically inhibit the formation of hydroxyl radicals, which is an important indicator of the body’s antioxidant capacity [34,35]. The total antioxidant capacity (T-AOC) is a comprehensive indicator used to measure the functional status of the body’s antioxidant system, reflecting the body’s antioxidant capacity [36]. In this study, pBD2 increased SOD and T-AOC activities, with no effect on MDA. In some studies, antimicrobial peptides significantly enhanced the antioxidant capacity of weaned piglets [37,38], which is consistent with our results. These results indicate that pBD2 enhanced the antioxidant capacity of weaned piglets.

Weaned piglets often have low immunity. Immunoglobulins and cytokines are important components of the immune system in resisting pathogen invasion. Immunoglobulins are small molecular proteins produced by B lymphocytes and mainly include IgA, IgG, and IgM. They are an important part of humoral immunity, binding to externally infected pathogens and eliminating them to effectively protect the body [39]. Pro-inflammatory cytokines are released in large amounts upon stimulation by external pathogens, causing inflammatory reactions and leading to tissue damage. IL-6 and TNF-α are common pro-inflammatory cytokines [40]. Anti-inflammatory cytokines inhibit the release of pro-inflammatory cytokines, reduce excessive inflammatory responses, and protect normal cells from destruction. IL-2 and IL-10 are well-studied anti-inflammatory cytokines [41]. IL-2 stimulates T-cell growth, proliferation, and differentiation and is an important immune enhancer [41]. IL-10 is a pleiotropic cytokine that inhibits the expression of inflammatory factors through activated macrophages [42]. This study measured the contents of immunoglobulins IgA, IgG, and IgM and cytokines IL-2, IL-10, IL-6, and TNF-α in serum. The results showed that pBD2 significantly increased IgM and IL-10 levels but had no effect on IgA, IgG, IL-2, IL-6, and TNF-α, indicating pBD2’s potential in improving immune function and reducing inflammation in weaned piglets. In some studies, antimicrobial peptides enhanced immune performance and disease resistance by affecting the contents of immunoglobulins and cytokines. Chicken intestinal antimicrobial peptides enhanced the contents of IgG and IgM in serum [43]. Antimicrobial peptide microcin C7 significantly increased the levels of serum cytokine IL-10 and immunoglobulins IgG and IgM in broilers. Additionally, some studies have shown that antimicrobial peptides can reduce the levels of pro-inflammatory cytokines IL-6 and TNF-α [44,45]. These results showed that different antimicrobial peptides might have different effects on immunoglobulins and cytokines, indicating their potential to improve immune function and reduce inflammation in weaned piglets. As this study only observed pBD2’s effects in the context of IL-10 and IgM, more immune markers need to be studied in further research.

The digestion and absorption of nutrients are closely related to the gut’s health status in weaned piglets, which, in turn, is influenced by factors such as gut morphology and the intestinal barrier. The intestinal barrier function is primarily maintained through the regulation of tight-junction proteins, which play a crucial role in optimizing gut health. Abnormal expression of tight-junction proteins can lead to increased paracellular permeability, contributing to intestinal and systemic diseases [46]. Antimicrobial peptides as feed additives, upon entering the animal gut, can accumulate in the jejunum and ileum, stimulating the proliferation of epithelial cells in these regions, thereby promoting villus growth and improving gut morphology [47]. In this study, pBD2 was found to increase the ratio of the villus length to crypt depth. In the duodenum and jejunum, the HP group significantly upregulated the expression of ZO-1 and claudin-1 while having no effect on occludin. Meanwhile, the LP group had no effect on TJs. In the ileum, the expression of TJs was not significant in either the HP or LP group compared with the control group. AMPs possessed intestinal homeostasis and intestinal barrier functions. Deficiency in AMPs may lead to barrier dysfunction and dysbiosis in the gut. AMPs have been shown to enhance mucosal barrier function by directly inducing the expression of TJ proteins. Dietary supplementation with AMPs has typically improved the intestinal morphology, production performance, and feed efficiency in livestock [48]. For instance, the oral administration of AMP buforin II increased the expression of claudin-1, occludin, and ZO-1 in the jejunum of piglets challenged with *Escherichia coli* [49]. AMP cathelicidin BF induces ZO-1 expression and restores LPS-mediated intestinal damage and barrier function dysfunction [50]. pBD2 has been found to restore the expression of MUC1, MUC2, claudin-1, ZO-1, and ZO-2, as well as the colonic barrier integrity, in DSS-treated mice [44]. Additionally, pBD2 restored the abnormal expression of ZO-1 and occludin in IPEC-J2 cells challenged with *E. coli* [14]. pBD2 enhanced the intestinal barrier by upregulating the expression of TJs in the duodenum and jejunum, similar to previous studies.

Dietary and environmental changes are significant factors influencing the microbial composition of the digestive system in piglets. After weaning, piglets undergo drastic changes in their dietary structure and living environment, leading to intestinal microbial dysbiosis. During this period, the piglets’ intestines become more vulnerable due to alterations in structure and barrier properties, potentially resulting in digestive disorders, diarrhea, growth retardation, and increased mortality. Maintaining a stable balance of intestinal microbiota is fundamental to ensuring good health in weaned piglets [51]. In this study, pBD2 was found to regulate the composition and abundance of intestinal microbiota in weaned piglets. In the LP group, several microorganisms exhibited an increasing trend, including *Prevotellaceae*, *Clostridium_sensu_stricto_*1, *Fusicatenibacter*, *Lachnospiraceae*, and *Megasphaera*. Prevotellaceae, which are crucial gut microbiota in animals, are known for their ability to degrade mucoproteins and plant polysaccharides, thereby enhancing the digestion, absorption, and utilization of nutrients from feed [52]. *Clostridium_sensu_stricto*_1 has been reported to produce short-chain fatty acids (SCFAs) and promote intestinal mucosal barrier function [53]. *Fusicatenibacter* can inhibit intestinal inflammation and is positively correlated with SCFA production [54]. *Lachnospiraceae* are closely related to pig health. Healthy pigs showed a higher abundance of *Lachnospiraceae* compared with weaned piglets with diarrhea [55]. Additionally, *Lachnospiraceae* may possess strong fiber-degrading capabilities [56]. *Megasphaera* is capable of producing all types of SCFAs [57]. Conversely, the abundance of uncultured_bacterium_g_*Bacteroidales_RF16_group*, *Muribaculaceae*, *Paludibacteraceae*, *Christensenellaceae*, uncultured_bacterium_g_*XBB*1006, *Ruminococcaceae_UCG_*005*, Eubacterium-coprostanoligens_group*, *Escherichia_Shigella*, *Treponema* 2, and *Spirochaetaceae* was decreased in the LP group compared with the control group. In a previous study, *Escherichia-Shigella* and *Ruminococcaceae_UCG-*005 were more abundant in weak piglets compared with healthy ones [58]. *Escherichia-Shigella* is considered to play a significant role in the development of diarrhea in piglets and can severely impact their intestinal barrier function [59]. Members of the *Spirochaetaceae* family, mostly pathogens or opportunistic pathogens, can potentially induce diarrhea in piglets [60]. These results indicate that the LP group enhanced intestinal nutrient digestion and reduced the abundance of harmful bacteria. In the HP group, the abundance of *Oscillospira* and *Prevotellaceae* was increased, and the abundance of *Spirochaeta*, *Treponema* 2, *Ruminococcaceae_UCG_*005, *Rikenellaceae_RC*9*_gut_group*, *Eubacterium_coprostanoligenes_group*, and *Mitsuokella* was decreased. Interestingly, these microorganisms had the same enrichment trend as the LP group, but with a reduced number of microbial taxa exhibiting altered abundance, except for *Mitsuokella*, which did not show significant changes in the LP group. These results indicate that different concentrations of pBD2 had varying degrees of influence on different microbials, and that the intestinal microbiota exhibited sensitivity to the dosage of pBD2. Additionally, in the correlation analysis, *Prevotella_9* showed a positive correlation with SOD and T-AOC; uncultured_bacterium_f_*Muribaculaceae*, *Ruminococcaceae_UCG-*005, and *Treponema_*2 showed a negative correlation with IgM and T-AOC; and *[Eubacterium]_coprostanoligenes_group* showed a negative correlation with IL-10, IgM, and T-AOC. pBD2 might affect the content of SOD, T-AOC, IgM, and IL-10 by increasing the abundance of *Prevotella_9* and decreasing the abundance of uncultured_bacterium_f_*Muribaculaceae*, *Ruminococcaceae_UCG-*005, *Treponema_*2, and *[Eubacterium]_coprostanoligenes_group*. pBD2 regulated the abundance of those microorganisms to enhance the immune performance and antioxidant capacity. Several studies have already confirmed the positive role of AMPs in modulating the intestinal microbiota [61], with the oral administration of defensins increasing the number of beneficial bacteria and reducing harmful bacteria such as *Escherichia coli* and *Streptococcus* [24,32], similar to the results of our study.

## 5. Conclusions

Dietary supplementation with pBD2 can improve growth performance, antioxidant capacity, and intestinal barrier function. pBD2 increased the contents of IL-10 and IgM; however, more immune markers need to be studied in future research. Additionally, pBD2 affects the abundance and composition of the intestinal microbiota, and this effect is dose-dependent. Low doses of pBD2 reduce the abundance of harmful bacteria associated with intestinal inflammation and diarrhea, as well as increasing the abundance of beneficial bacteria related to nutrient and energy metabolism. pBD2 may affect antioxidant capacity and immune function by regulating the intestinal microbiota. These results suggest that pBD2 has the potential to replace antibiotics as a novel feed additive in production.

## Figures and Tables

**Table 1:** Composition and nutritional composition of basic diet.

Feed Composition	Content (%)
Corn	22.78
Broken rice	20.00
Expanded corn	12.0
Expanded soybean	9.00
Soybean meal	13.00
Soybean protein concentrate	2.00
Whey powder	4.00
Fish meal	4.00
Fermented soybean meal	6.00
Soybean oil	2.00
White sugar	2.00
Salt	0.27
DL methionine	0.17
L-lysine (98%)	0.20
Threonine (98.5%)	0.09
Tryptophan (98%)	0.02
Calcium hydrogen phosphate	0.92
Stone powder	0.55
Premix ^1^	1.00
Total	100
Nutritional level	
(Kcal/kg)	2660
Crude protein (%)	20.79
Calcium (%)	0.62
Available phosphorus (%)	0.37
Lysine (%)	1.32
Threonine (%)	0.88
Methionine + cysteine (%)	0.84

^1^ Containing per kilogram of supplement: 12,000 IU of vitamin A, 3000 IU of vitamin D3, 20 mg of vitamin E, 2 mg of vitamin K3, 4 mg of vitamin B1, 3.6 mg of vitamin B2, 4 mg of vitamin B6, 0.02 mg of vitamin B12, 0.15 mg of biotin, 1.0 mg of folic acid, 11 mg of *D*-pantothenic acid, 10 mg of nicotinic acid, 120 mg of copper, 80 mg of iron, 80 mg of manganese, 1600 mg of zinc, 0.40 mg of iodine, 0.30 mg of selenium, 500 g of feeding attractant, 2000 IU of phytase, and 600 mg of choline.

**Table 2:** Primers used for quantitative real-time PCR.

Gene Name	Primer Sequence (5′→3′)	GenBank Number	Product Length
ZO-1	Forward: AGCCCGAGGCGTGTTT Reverse: GGTGGGAGGATGCTGTTG	XM_021098896.1	147 bp
Claudin-1	Forward: ATTTCAGGTCTGGCTATCTTAGTTGC Reverse: AGGGCCTTGGTGTTGGGTAA	NM_001244539.1	214 bp
Occludin	Forward: ATCAACAAAGGCAACTCT Reverse: GCAGCAGCCATGTACTCT	NM_001163647.2	157 bp
GAPDH	Forward: ATGGTGAAGGTCGGAGTGAA Reverse: CGTGGGTGGAATCATACTGG	NM_001206359.1	154 bp

**Table 3:** Effects of pBD2 on growth performance of weaned piglets.

Index	Control	LP	HP	SEM	*p*-Value
Initial weight (kg)	6.18	6.38	6.21	0.11	0.79
Total weight gain (kg)	1.45 ^b^	1.95 ^a^	2.24 ^a^	0.13	0.02
ADG (kg)	0.10 ^b^	0.14 ^a^	0.16 ^a^	0.01	0.02
ADFI (g)	198.58	256.04	285.91	17.14	0.11
FCR	0.52	0.54	0.59	0.02	0.42

n = 5 for each treatment. ^a,b^ means in the same row with different superscripts are significantly different (*p*-value < 0.05). Control, regular diet. LP, basal diet supplemented with 2.5 mg/kg of pBD2. HP, basal diet supplemented with 5 mg/kg of pBD2.

**Table 4:** Effects of pBD2 on antioxidant properties of weaned piglets.

Index	Control	LP	HP	SEM	*p*-Value
T-AOC (μmol/mL)	2.44 ^b^	3.67 ^a^	3.31 ^a^	0.20	0.04
SOD (U/mL)	78.74 ^b^	212.37 ^a^	167.38 ^b^	24.45	0.05
MDA (nmol/mL)	8.16	8.89	6.88	0.57	0.45

n = 5 for each treatment. ^a,b^ means in the same row with different superscripts are significantly different (*p*-value < 0.05). Control, regular diet. LP, basal diet supplemented with 2.5 mg/kg of pBD2. HP, basal diet supplemented with 5 mg/kg of pBD2.

**Table 5:** Effects of pBD2 on immune performance of weaned piglets.

Index	Control	LP	HP	SEM	*p*-Value
IgA (μg/mL)	639.02	654.47	646.64.97	7.76	0.27
IgM (mg/mL)	14.00 ^b^	15.26 ^a^	15.37 ^a^	0.27	0.033
IgG (mg/mL)	18.72	18.47	19.84	0.33	0.214
IL-2 (pg/mL)	265.08	298.21	294.33	6.77	0.066
IL-10 (pg/mL)	356.16 ^b^	375.28 ^ab^	400.09 ^a^	8.00	0.05
IL-6 (pg/mL)	80.22	82.88	88.39	1.80	0.17
TNF-α (pg/mL)	494.48	498.87	510.34	8.78	0.784

n = 5 for each treatment. ^a,b^ means in the same row with different superscripts are significantly different (*p*-value < 0.05). Control, regular diet. LP, basal diet supplemented with 2.5 mg/kg of pBD2. HP, basal diet supplemented with 5 mg/kg of pBD2.

**Table 6:** Effect of pBD2 on villus-to-crypt ratios of intestinal tissues.

Intestinal Tissue	Index	Control	LP	HP	SEM	*p*-Value
Duodenum	Villus (μm)	314.15	330.30	336.86	3.01	0.18
	Crypt (μm)	151.46	144.05	137.69	6.92	0.41
	C/V	2.08 ^b^	2.30 ^a^	2.46 ^a^	0.059	0.023
Jejunum	Villus (μm)	232.39 ^c^	309.75 ^b^	368.31 ^a^	16.10	0.00
	Crypt (μm)	151.26	151.84	141.42	4.76	0.63
	C/V	1.59 ^b^	2.05 ^a^	2.60 ^a^	0.12	0.00
Ileum	Villus (μm)	278.26	286.89	317.22	11.00	0.34
	Crypt (μm)	132.81	123.36	118.51	3.41	0.23
	C/V	2.10 ^c^	2.34 ^b^	2.67 ^a^	0.085	0.013

n = 5 for each treatment. ^a–c^ means in the same row with different superscripts are significantly different (*p*-value < 0.05). Control, regular diet. LP, basal diet supplemented with 2.5 mg/kg of pBD2. HP, basal diet supplemented with 5 mg/kg of pBD2.

**Table 7:** Analysis of α-diversity.

Index	Control	LP	HP	SEM	*p*-Value
ACE	596.94 ^a^	580.69 ^ab^	550.7 ^b^	8.08	0.026
Chao1	605.29	584.39	571.18	7.19	0.143
Simpson	0.98 ^a^	0.98 ^a^	0.96 ^b^	0.004	0.023
Shannon	6.9 ^a^	7.01 ^a^	6.05 ^b^	0.16	0.005

n = 5 for each treatment. ^a,b^ means in the same row with different superscripts are significantly different (*p*-value < 0.05). Control, regular diet. LP, basal diet supplemented with 2.5 mg/kg of pBD2. HP, basal diet supplemented with 5 mg/kg of pBD2.

## Data Availability

The 16S rRNA sequence datasets are publicly available through NCBI’s Sequence Read Archive, under accession number PRJNA1262633.
